# A Selenium‐Enriched Composite From Edible Mushrooms and Medicinal Plants Ameliorates Metabolic Disorders via Multi‐Target Network Modulation

**DOI:** 10.1002/fsn3.72569

**Published:** 2026-11-09

**Authors:** Wuxian Zhou, Fanfan Wang, Yuanyuan Duan, Xiaogang Jiang, Hongchang Xiao, Aimin Yu, Hui Kuang, Bo Zhu, Qingfang Wang, Jingmao You

**Affiliations:** ^1^ Key Laboratory of Biology and Cultivation of Herb Medicine, Ministry of Agricultural and Rural Affairs Institute of Chinese Herbal Medicines, Hubei Academy of Agricultural Sciences Enshi China; ^2^ Zhejiang Key Laboratory of Chinese Medicine Modernization School of Pharmaceutical Sciences, Zhejiang Chinese Medical University Hangzhou China

**Keywords:** edible mushrooms, glucolipid metabolism, hyperuricemia, medicinal plants, molecular docking, multi‐target modulation, network pharmacology, selenium‐enriched composite

## Abstract

Inspired by the dietary habits of centenarians in Enshi, China, this study developed two selenium‐enriched water‐soluble powder formulations (OFXBJC and NFXBJC) from edible mushrooms and medicinal plants. Comparison of their active components and metabolomic profiles showed that NFXBJC contained 39.0% higher total flavonoids, 33.4% higher total saponins, and 38.6% higher soluble protein, along with upregulated hypoglycemic metabolites. NFXBJC was therefore selected for subsequent evaluation of its effects on glucolipid disorders, renal injury, and hyperuricemia. In a Type 2 diabetic mouse model, low‐dose NFXBJC (80 mg/kg/day) dose‐dependently increased serum selenium levels, reduced fasting and postprandial blood glucose by 36.2% and 38.9%, respectively, lowered total cholesterol and triglycerides by 14.8% and 35.6%, respectively, and restored renal function without obvious organ toxicity. Correlation analysis revealed a strong negative association between blood glucose and serum selenium (*R* ^2^ = 0.964). Network pharmacology identified 48 candidate compounds and 552 putative targets, with the top 10 hub targets (STAT3, IL6, AKT1, CTNNB1, TNF, EGFR, HIF1A, IL1B, SRC, and BCL2) fully conserved across diabetes, hyperlipidemia, and hyperuricemia, suggesting multi‐target modulation of a shared inflammation–metabolism network across these three conditions. Molecular docking further predicted stable binding of core active ingredients (e.g., cryptotanshinone, isorhamnetin, kaempferol) to these hub targets, with binding energies below −5.0 kcal/mol. An exploratory small‐scale human observational study suggested that NFXBJC may progressively reduce uric acid (32.2%–39.8%), blood glucose, and total cholesterol. Collectively, NFXBJC exerts beneficial effects on glucolipid metabolism, renal function, selenium status, and uric acid levels via multi‐target mechanisms, supporting its potential as a functional food for metabolic health.

## Graphical abstract

Inspired by the dietary habits of centenarians in Enshi, China, two selenium‐enriched water‐soluble powder formulations (OFXBJC and NFXBJC) were developed from edible mushrooms and medicinal plants. NFXBJC exhibited higher bioactive components and hypoglycemic metabolites, and was selected for subsequent evaluations. In a Type 2 diabetic mouse model, NFXBJC significantly ameliorated glucolipid metabolism and renal function, accompanied by a strong negative correlation between blood glucose and serum selenium. Network pharmacology and molecular docking identified 10 conserved hub targets (e.g., STAT3, IL6, AKT1) bridging inflammation and metabolism across diabetes, hyperlipidemia, and hyperuricemia, revealing the multi‐target mechanisms of its core active compounds. An exploratory human observation further preliminarily validated its efficacy in reducing uric acid, blood glucose, and total cholesterol. These findings support NFXBJC as a promising functional food for metabolic health.

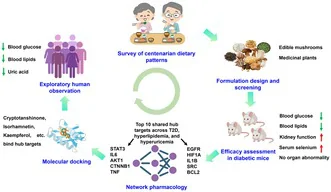

## 1 Introduction

With shifts in modern lifestyle, the prevalence of glucolipid metabolism‐related disorders continues to rise. Diabetes, hyperlipidemia, and hyperuricemia have become major chronic diseases threatening public health (Fang et al. 2024; Ma et al. 2025). Although first‐line clinical hypoglycemic agents such as metformin have proven efficacy, long‐term use may be associated with adverse effects including gastrointestinal discomfort and hepatorenal damage (Diabetes Prevention Program Research Group 2012). Therefore, the development of safe, effective, natural functional foods suitable for long‐term consumption is of great importance for the prevention and adjuvant treatment of metabolic diseases. Plant‐derived bioactive compounds, such as phenolics, flavonoids, and terpenoids, have shown potential in functional food and nutraceutical formulations, with studies demonstrating their antioxidant, anti‐mutagenic, and protective activities (Yasir et al. 2022).

Traditional Chinese medicine has long embraced the concept of “medicine and food homology” (Liu et al. 2026). Our research originated from observations of the longevity phenomenon in Enshi City, known as the “Selenium Capital of the World.” During fieldwork in Jianshi County, we visited multiple healthy centenarians and found that their regular diet included various local wild mushrooms (e.g., *Agaricus blazei*, *Cordyceps militaris*, *Lentinula edodes*, *Hericium erinaceus*, *Tremella fuciformis*, *Morchella esculenta*, *Flammulina filiformis*, *Pleurotus eryngii*, *Pleurotus ostreatus*, and *Boletus edulis*), as well as medicinal plants including *Morus alba* , *Panax ginseng*, *Ampelopsis grossedentata* (vine tea), *Cyclocarya paliurus*, and *Polygonatum sibiricum*. Notably, these centenarians had a low prevalence of chronic diseases such as hypertension and diabetes despite their advanced age.

Accumulating evidence indicates that all the aforementioned ingredients possess diverse biological activities. For example, mushroom polysaccharides exhibit immunomodulatory and hypoglycemic effects (Zhang et al. 2026); alkaloids in *Morus alba* inhibit α‐glucosidase activity (Ji et al. 2016); triterpenoids and flavonoids in *Cyclocarya paliurus* exert significant hypoglycemic activity and are known as “plant insulin” (Sun et al. 2020); steroidal saponins in *Polygonatum sibiricum* exhibit hypoglycemic and renoprotective effects (Zhang et al. 2023); and *Ampelopsis grossedentata* is rich in flavonoids with antioxidant and lipid‐lowering activities (Zhou et al. 2022). Selenium is an essential micronutrient for humans, and its deficiency is closely associated with hyperglycemia, dyslipidemia, and hyperuricemia (Navarro‐Alarcon et al. 1999; Bleys et al. 2007). Furthermore, organic selenium compounds have been shown to exert anti‐hyperglycemic effects by modulating redox and insulin signaling (Dos Santos et al. 2020). Preliminary analyses indicated that most of these raw materials meet the selenium‐enriched standard (≥ 0.2 mg/kg) (Zhou et al. 2021), suggesting that local residents may obtain natural organic selenium through the food chain (Huang et al. 2013).

Guided by modern nutritional science and traditional Chinese medicine theory (Tian et al. 2026), two selenium‐enriched water‐soluble powder products (OFXBJC and NFXBJC) were developed using locally sourced mushrooms, medicinal plants, and selenium‐enriched broccoli from Enshi. The two formulations were designed based on two representative dietary patterns identified among Enshi centenarians during field investigations. The first pattern featured a higher proportion of edible mushrooms combined with *Morus alba* , *Panax ginseng*, and *Ampelopsis grossedentata*; the second pattern was characterized by higher proportions of *Cyclocarya paliurus* and *Polygonatum sibiricum* paired with a similar set of mushrooms. These two patterns formed the basis for OFXBJC and NFXBJC, respectively. Each ingredient was selected for its complementary bioactive components: mushrooms provide polysaccharides and soluble proteins; *Morus alba* , *Cyclocarya paliurus*, *Polygonatum sibiricum*, *Ampelopsis grossedentata*, and *Panax ginseng* provide alkaloids, flavonoids, triterpenoids, and steroidal saponins with hypoglycemic, hypolipidemic, and renoprotective activities; and selenium‐enriched broccoli provides organic selenium. Ingredient proportions were determined based on traditional consumption ratios observed in the Enshi centenarian diet, measured selenium contents of raw materials, and literature‐reported effective doses of key bioactive compounds. This multi‐component, selenium‐enriched design distinguishes these products from conventional single‐target functional foods and is intended to simultaneously modulate glucolipid metabolism, renal function, and uric acid levels via multi‐target network regulation (Wang, Hu, et al. 2024; Wang, Liu, et al. 2024; Wang, Li, et al. 2024; Liu et al. 2025).

This study compared the active components and metabolomic profiles of the two formulations (OFXBJC and NFXBJC), and the optimized formulation NFXBJC was selected for subsequent efficacy evaluations. Using a Type 2 diabetic mouse model induced by streptozotocin combined with a high‐fat diet, we evaluated the effects of NFXBJC on blood glucose, blood lipids, body weight, renal function, and organ histomorphology, and analyzed correlations among these indicators. Finally, an exploratory small‐scale human observational study was conducted to preliminarily assess the potential effects of NFXBJC on individuals with hyperglycemia, hyperlipidemia, and/or hyperuricemia, aiming to provide a scientific basis for NFXBJC as an adjuvant functional food for improving glucolipid metabolism and renal function.

## 2 Materials and Methods

### 2.1 Test Materials

Two selenium‐enriched water‐soluble powder formulations, OFXBJC and NFXBJC, were prepared from edible mushrooms and medicinal plants. The raw materials included dried mushrooms (*Agaricus blazei*, *Cordyceps militaris*, *Lentinula edodes*, *Hericium erinaceus*, *Tremella fuciformis*, *Morchella esculenta*, *Flammulina filiformis*, *Pleurotus eryngii*, *Pleurotus ostreatus*, and *Boletus edulis*) and plants (selenium‐enriched broccoli, *Morus alba* , *Panax ginseng*, *Ampelopsis grossedentata*, *Cyclocarya paliurus*, and *Polygonatum sibiricum*). These raw materials were extracted with hot water, and the resulting extracts were compounded in different proportions to produce OFXBJC and NFXBJC (Table 1). The elemental selenium contents of OFXBJC and NFXBJC were 30.0 mg/kg and 37.4 mg/kg, respectively.

**TABLE 1:** Raw material compositions of the two formulations, OFXBJC and NFXBJC.

No.	OFXBJC		NFXBJC	
	Materials	Proportion (%)	Materials	Proportion (%)
1	*Agaricus blazei*	3	*Agaricus blazei*	2
2	*Cordyceps militaris*	3	*Cyclocarya paliurus*	13
3	*Lentinula edodes*	7	*Lentinula edodes*	13.5
4	*Hericium erinaceus*	8	*Hericium erinaceus*	2
5	*Tremella fuciformis*	9	*Tremella fuciformis*	5
6	*Morchella esculenta*	3	*Morchella esculenta*	2
7	*Flammulina filiformis*	8	*Flammulina filiformis*	13
8	*Pleurotus eryngii*	3	*Pleurotus eryngii*	2
9	*Pleurotus ostreatus*	8	*Pleurotus ostreatus*	2
10	*Boletus edulis*	8	*Boletus edulis*	13
11	*Selenium‐enriched broccoli*	12.5	*Selenium‐enriched broccoli*	8
12	*Morus alba*	12.5	*Morus alba*	12.5
13	*Panax ginseng*	5	*Polygonatum sibiricum*	2
14	*Ampelopsis grossedentata*	10	*Ampelopsis grossedentata*	10

The hot water extraction procedure was as follows: raw materials were crushed, mixed with 10–20 volumes of water, and heated at 80°C–100°C to obtain the extract solution. The extract was then ultrafiltered at 60°C–80°C through a composite filter membrane (diatomaceous earth, activated carbon, and titanium dioxide coated on a polyvinylidene fluoride membrane) to obtain a crude filtrate. The crude filtrate was further refined using a 100–200 nm ceramic membrane at 20°C–50°C, followed by ultrafiltration through a 2–10 kDa molecular weight cutoff membrane at 30°C–50°C. The resulting ultrafiltrate was spray‐dried to obtain the raw material extract. The extraction yields of individual raw materials ranged from 3.0% to 28.0% (w/w, dry basis), and the final yields of OFXBJC and NFXBJC were 12.6% and 12.2% (w/w, dry basis), respectively. Detailed extraction yields for each raw material are provided in Table S1.

### 2.2 Experimental Design

#### 2.2.1 Evaluation of Glucolipid Metabolism and Renal Function in Mice

A total of 40 eight‐week‐old male C57BL/6 mice were housed in a specific pathogen‐free animal facility under controlled conditions (temperature 22°C ± 2°C, humidity 50% ± 10%, 12 h light/12 h dark cycle, ad libitum access to food and water). After one week of acclimatization, mice were randomly divided into five groups (*n* = 8 per group): normal control group (CK), STZ‐induced diabetes model group (STZD), positive control metformin group (STZM, 200 mg/kg/day), low‐dose NFXBJC group (STZL, 80 mg/kg/day), and high‐dose NFXBJC group (STZH, 400 mg/kg/day). Allocation concealment was ensured: a researcher not involved in subsequent data analysis generated the randomization sequence and assigned group allocations. Investigators performing animal experiments, biochemical measurements, and histopathological assessments were blinded to group assignments. Group codes were revealed only after all data collection and analysis were completed.

Except for the normal control group, all groups were fed a high‐fat diet for 4 weeks and received intraperitoneal injections of streptozotocin (STZ, 50 mg/kg) for 5 consecutive days to establish the Type 2 diabetes model. NFXBJC and metformin were administered daily by gavage from Day 25 until the end of the experiment on Day 50. During the experiment, fasting blood glucose (FBG), postprandial blood glucose (PBG), and body weight were measured. At the experimental endpoint, serum renal function indicators (urea, creatinine) and blood lipid indicators (total cholesterol [TC], triglycerides [TG]) were measured, and histopathological changes in the liver, kidney, spleen, and pancreas were examined (Figure 1). All animal procedures were approved by the Laboratory Animal Welfare and Ethics Committee of the Institute of Chinese Herbal Medicines, Hubei Academy of Agricultural Sciences (Approval No. ICHM‐A‐2025002).

**FIGURE 1:**
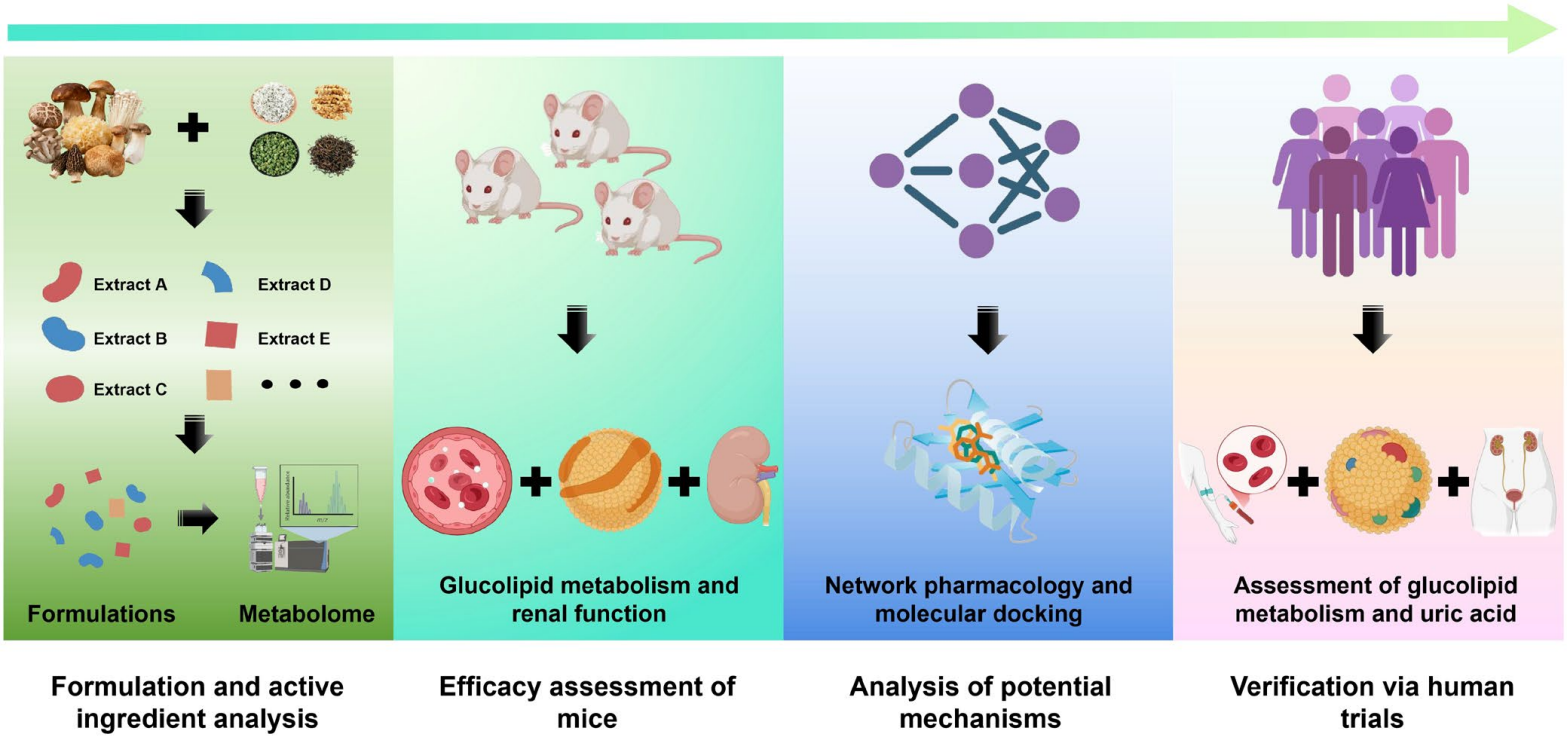
Flowchart of the development and efficacy study of the NFXBJC formulation. Extract A–E represent hot‐water extracts prepared from different raw materials (edible mushrooms and medicinal plants), and the ellipsis indicates additional extracts not individually labeled. These extracts were compounded in different proportions to produce two candidate formulations, OFXBJC and NFXBJC, followed by active component determination and metabolomic profiling. The optimized formulation NFXBJC was then selected for efficacy assessment in mice, network pharmacology and molecular docking analysis, and exploratory human observation.

#### 2.2.2 Evaluation of Glucolipid Metabolism and Uric Acid in Humans

To evaluate the effects of NFXBJC on fasting blood glucose (FBG), total cholesterol (TC), and uric acid (UA) levels in humans, four subjects were enrolled, all of whom had at least one of these indicators above the normal upper limit. They resided in the same residential area and maintained consistent dietary and daily routines throughout the study period. Exclusion criteria included severe heart, liver, or kidney diseases, malignant tumors, mental illness, use of glucose‐, lipid‐, or uric acid‐lowering medications within the previous 3 months, and allergy to any component of the product. Three subjects (groups X, Y, and Z) consumed 2 g of NFXBJC dissolved in 200 mL of warm water three times daily after meals, corresponding to a daily intake of 6 g NFXBJC and approximately 224.4 μg of elemental selenium per day. The remaining subject (group W) served as the control and received a maltodextrin‐based placebo at the same frequency.

Given the exploratory and small‐scale nature of this study, randomization, allocation concealment, and participant blinding were not applied. To minimize expectation bias, the placebo was formulated to match NFXBJC in appearance, taste, and packaging. All biochemical measurements were performed using a portable detector, and data were analyzed by a researcher not involved in the intervention. No participants consumed other supplements or medications that might affect glucolipid metabolism during the study. All subjects recorded their daily diet and physical symptoms. Changes in blood glucose, blood lipids, and UA were measured at baseline (Day 0) and on Days 15, 30, 45, and 120 after the start of administration. At each time point, each indicator was measured in quadruplicate for every subject, and the mean value was used for analysis. Subject baseline characteristics are summarized in Table 2. This study was not registered as a clinical trial and was approved by the Human Research Ethics Committee of the Institute of Chinese Herbal Medicines, Hubei Academy of Agricultural Sciences (Approval No. ICHM‐H‐2025001).

**TABLE 2:** Baseline characteristics of the subjects in the exploratory human observation.

Subject ID	Age	Gender	BMI (kg/m^2^)	Body weight (kg)	FBG (mmol/L)	HbA1c (%)	TC (mmol/L)	UA (μmol/L)	Medication	Dietary habits
W	35	Female	22.5	55	5.2	5.4	4.8	390.50	None	Regular
X	62	Female	24.8	62	5.8	5.9	5.1	425.25	None	Regular
Y	63	Male	27.3	70	6.75	7.2	9.60	460.50	None	Regular
Z	35	Male	23.5	68	5.5	5.6	4.9	601.75	None	Regular

*Note:* Normal levels of relevant indicators in adults are: fasting blood glucose (FBG) 3.9–6.1 mmol/L, total cholesterol (TC) < 5.2 mmol/L, uric acid (UA) 202–416 μmol/L for males and 142–360 μmol/L for females.

### 2.3 Determination of Active Components

Total flavonoid content in OFXBJC and NFXBJC was determined by the AlCl_3_ colorimetric method using rutin as the standard (detection wavelength 415 nm) (Magalhães et al. 2012). Total saponin content was determined by the perchloric acid colorimetric method using diosgenin as the standard (detection wavelength 408 nm) (de Aguiar et al. 2024). Polysaccharide content was determined by the anthrone‐sulfuric acid method using anhydrous glucose as the standard (detection wavelength 580 nm) (Hao et al. 2018). Soluble protein content was determined by the Coomassie Brilliant Blue G‐250 method using bovine serum albumin as the standard (detection wavelength 595 nm) (Yang et al. 2021).

### 2.4 Metabolomics Analysis

Untargeted metabolomics analysis of OFXBJC and NFXBJC samples was performed using ultra‐performance liquid chromatography–tandem mass spectrometry (UPLC–MS/MS) (Salem et al. 2020). The dried extracts were reconstituted in pre‐cooled (4°C) 70% aqueous methanol containing internal standards by vortexing, followed by centrifugation and filtration prior to LC–MS/MS analysis. Chromatographic separation was performed on a Waters ACQUITY UPLC HSS T3 column (1.8 μm, 2.1 mm × 100 mm) at 40°C, with gradient elution using 0.1% formic acid in water and acetonitrile as the mobile phase. Mass spectrometry was performed in positive and negative ion modes using an AB TripleTOF 6600 system. Raw data were processed by XCMS for peak extraction, alignment, and retention time correction. Metabolites were identified by searching in‐house and public databases, and peak areas were obtained for subsequent analysis.

### 2.5 Biochemical and Histological Analyses

Mouse FBG and PBG were measured in tail vein blood samples (Wang et al. 2026). Body weight changes were monitored using an electronic balance. At the experimental endpoint, blood was collected by orbital puncture to obtain serum. Serum urea, creatinine, TC, and TG were measured using an automatic biochemical analyzer to assess renal function and blood lipid profiles (Ndukaku et al. 2015; Hamzah 2019). Serum total selenium levels were determined by inductively coupled plasma mass spectrometry (ICP‐MS) according to established protocols (Sieniawska et al. 1999). Liver, kidney, spleen, and pancreas tissues were embedded in paraffin, sectioned, and stained with hematoxylin and eosin (H&E) for histological observation by light microscopy (Haligur et al. 2012). Histopathological changes were semi‐quantitatively scored by an observer blinded to group allocation using a 0–3 scale (0 = normal, 1 = mild, 2 = moderate, 3 = severe) as previously described (Landmann et al. 2021). The following parameters were scored: hepatocellular steatosis, inflammatory cell infiltration, hepatocyte swelling/degeneration, renal tubular injury, glomerular abnormalities, splenic white pulp hyperplasia, red pulp congestion, pancreatic islet atrophy, and acinar cell damage. For human samples, blood glucose, blood lipids, and UA were measured using a three‐in‐one portable detector (Cofoe, Kofu Medical Technology Co. Ltd.).

### 2.6 Network Pharmacology Analysis

To elucidate the potential mechanisms underlying the beneficial effects observed in the exploratory human observation, we performed network pharmacology analysis based on human disease targets. Candidate bioactive compounds were identified via untargeted metabolomic profiling of NFXBJC and validated against previously reported active ingredients in the literature. These compounds were screened using the Traditional Chinese Medicine Systems Pharmacology Database and Analysis Platform (TCMSP, https://www.tcmsp‐e.com/load_intro.php) with oral bioavailability (OB) ≥ 20% and drug‐likeness (DL) ≥ 0.15 as criteria. The DL threshold of 0.15 was adopted based on previous network pharmacology studies to ensure favorable drug‐likeness while retaining sufficient coverage of potential active ingredients (Chen et al. 2026). Corresponding targets were predicted via the Swiss Target Prediction database (http://swisstargetprediction.ch/) with a probability threshold > 0.1, and the resulting targets were standardized to official gene symbols.

Disease‐associated genes for Type 2 diabetes, hyperlipidemia, and hyperuricemia were retrieved from the GeneCards database (https://www.genecards.org/). Overlaps between compound targets and disease genes were visualized as Venn diagrams using the R packages VennDiagram and grid. For functional characterization, intersecting targets were submitted to the DAVID database (https://david.ncifcrf.gov/) for Gene Ontology (GO) and Kyoto Encyclopedia of Genes and Genomes (KEGG) pathway enrichment analyses with *Homo sapiens* as the reference background; results were visualized using the Bioinformatics online platform (http://www.bioinformatics.com.cn/). Protein–protein interaction (PPI) networks were constructed by uploading compound–disease intersection targets to the STRING database (https://cn.string‐db.org/). The resulting TSV files were imported into Cytoscape 3.8.2, and for each disease, the top 30 targets ranked by degree centrality within the intersection set were selected for network construction and visualization.

### 2.7 Molecular Docking

To further predict the binding affinity between core active compounds and hub targets identified from the PPI network, molecular docking was performed using AutoDock Vina (MGLTools‐1.5.7, OpenBabel‐2.3.2). Three‐dimensional structures of compounds and target proteins were retrieved from PubChem (https://pubchem.ncbi.nlm.nih.gov/) and the RCSB Protein Data Bank (PDB, https://www.rcsb.org/), respectively. Prior to docking, protein structures were prepared by removing water molecules and adding polar hydrogens, and compound structures were energy‐minimized. Core active ingredients were docked against the top 10 intersecting targets shared across diabetes, hyperlipidemia, and hyperuricemia, with a candidate target probability threshold ≥ 95%. Only compound–target pairs with predicted binding energies below −5.0 kcal/mol were retained for further analysis to ensure a reliable energetic criterion for potential bioactivity. Binding conformations with the lowest docking scores were selected as the most favorable poses, and results were visualized using PyMOL (version 3.0.3).

### 2.8 Data Analysis

Statistical analyses were performed using R software. Measurement data are expressed as mean ± standard deviation (SD). Normality was assessed using the Shapiro–Wilk test; data did not significantly deviate from normality, supporting the use of parametric tests. One‐way analysis of variance (ANOVA) was used for multiple group comparisons, followed by Tukey's HSD test for post hoc pairwise comparisons. Repeated measures ANOVA was used for time‐series data. Correlation analysis was performed using Pearson or Spearman correlation coefficients. Metabolomics data were analyzed by principal coordinates analysis (PCoA) based on Bray–Curtis distances to visualize overall differences in metabolite profiles between OFXBJC and NFXBJC. Differential metabolites were screened using variable importance in projection (VIP) > 1 and fold change ≥ 2 or ≤ 0.5 (|log_2_FC| ≥ 1) (Fan et al. 2016). A two‐sided *p* < 0.05 was considered statistically significant. Graphs were plotted using R packages including ggplot2 and pheatmap.

## 3 Results and Analysis

### 3.1 Comparison of Active Components

Compared with OFXBJC, NFXBJC showed no significant difference in polysaccharide content (*p* > 0.05), but had significantly higher total flavonoid (+39.0%), total saponin (+33.4%), and soluble protein (+38.6%) contents (all *p* < 0.05) (Figure 2), indicating that NFXBJC contains higher levels of bioactive components than OFXBJC.

**FIGURE 2:**
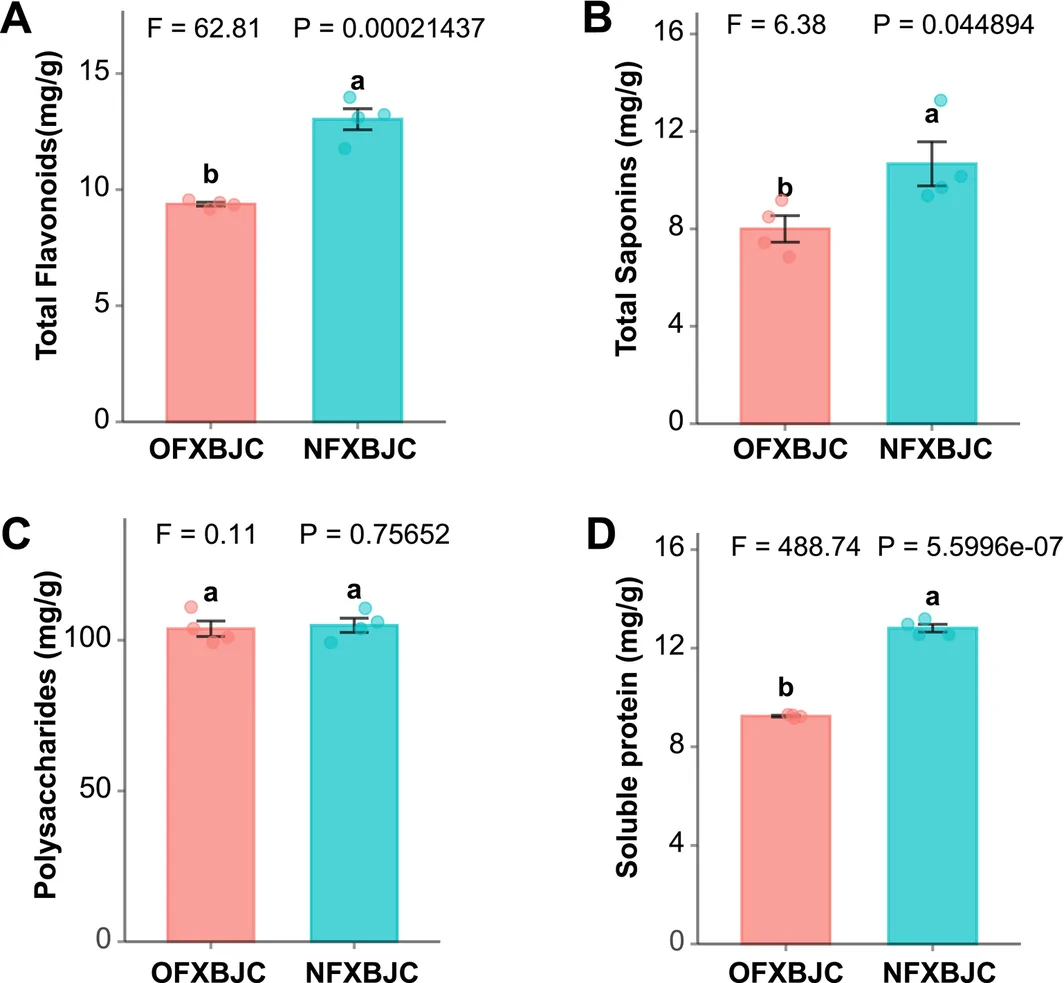
Comparison of active components between OFXBJC and NFXBJC. (A) Total flavonoid content; (B) Total saponin content; (C) Polysaccharide content; (D) Soluble protein content. Different letters indicate significant differences between groups (*p* < 0.05), while the same letter indicates no significant difference. Data are expressed as mean ± SD (*n* = 4).

### 3.2 Metabolomic Comparison Between the Two Candidate Formulations

To explore overall differences in metabolite profiles between the two candidate formulations, untargeted metabolomics analysis was performed. As shown in Figure 3A, PCoA based on Bray–Curtis distances showed that PCoA1 and PCoA2 explained 87.7% and 3.3% of the total variance, respectively. NFXBJC and OFXBJC samples were clearly separated along the PCoA1 axis, with OFXBJC clustering on the right and NFXBJC on the left. Adonis analysis confirmed that this separation was statistically significant (*R* ^2^ = 0.876, *p* = 0.022). The clustering heatmap (Figure 3B) further verified this separation, with the two groups forming distinct branches and showing good intragroup reproducibility. Most metabolites in NFXBJC showed an upregulation trend.

**FIGURE 3:**
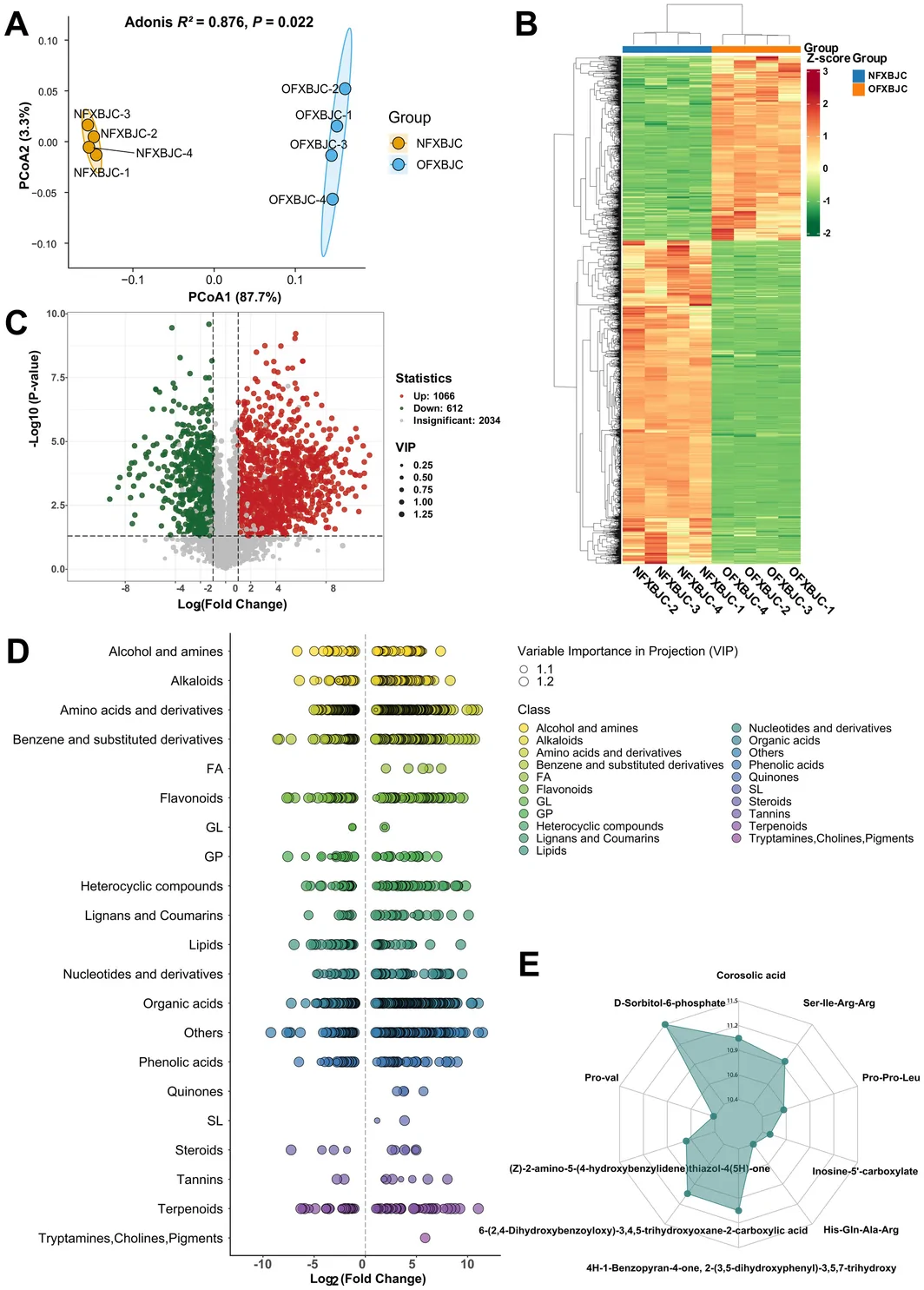
Metabolomic comparison between OFXBJC and NFXBJC. (A) PCoA score plot; (B) Clustering heatmap; (C) Volcano plot of differential metabolites; (D) Classification scatter plot of differential metabolites; (E) Names and expression changes of representative differential metabolites.

Differential metabolites were screened using the criteria VIP > 1 and |log_2_FC| ≥ 1. The volcano plot (Figure 3C) showed that, compared with OFXBJC, NFXBJC had 1066 upregulated and 612 downregulated metabolites, with upregulated metabolites predominating. The classification scatter plot (Figure 3D) showed that metabolites from various categories were distributed in both products. Flavonoids, terpenoids, and alkaloids were more abundant in the positive log_2_FC region, indicating that these components were generally increased in NFXBJC. VIP analysis further confirmed these results.

Detailed analysis of differential metabolites (Figure 3E) revealed that representative active metabolites with significantly increased content in NFXBJC included corosolic acid (with hypoglycemic activity), D‐sorbitol‐6‐phosphate, the dipeptide Pro‐Val, and various flavonoids such as 4H‐1‐benzopyran‐4‐one and 2‐(3,5‐dihydroxyphenyl)‐3,5,7‐trihydroxy. In contrast, relatively few metabolites showed significantly decreased content, mainly oligopeptides such as His‐Gln‐Ala‐Arg. Taken together, the metabolite composition of NFXBJC differed substantially from that of OFXBJC, with markedly higher contents of various metabolites with potential hypoglycemic and lipid‐lowering activities.

### 3.3 Effects of NFXBJC on Blood Glucose, Serum Selenium, Body Weight, and Postprandial Hyperglycemia Incidence in Mice

As shown in Figure 4A,B, the STZD group exhibited significantly elevated FBG and PBG compared with the CK group (*p* < 0.001), confirming successful diabetes induction. All treatment groups significantly reduced FBG and PBG. Compared with the STZD group, the STZL group reduced FBG by 36.2% (95% CI = 3.31–7.53 mmol/L, *p* < 0.001) and PBG by 38.9% (95% CI = 5.28–10.82 mmol/L, *p* < 0.001), an effect comparable to that of the metformin group (STZM). Regarding postprandial hyperglycemia (PBG ≥ 16.7 mmol/L), the heatmap (Figure 4E) showed that the STZD group maintained a high incidence (> 86%) throughout the experiment. The STZM and STZL groups showed marked reductions after treatment initiation (Day 25), with the STZL group reaching the lowest endpoint incidence (13%) and even achieving 0% on Day 37, followed by the STZH group (38%).

**FIGURE 4:**
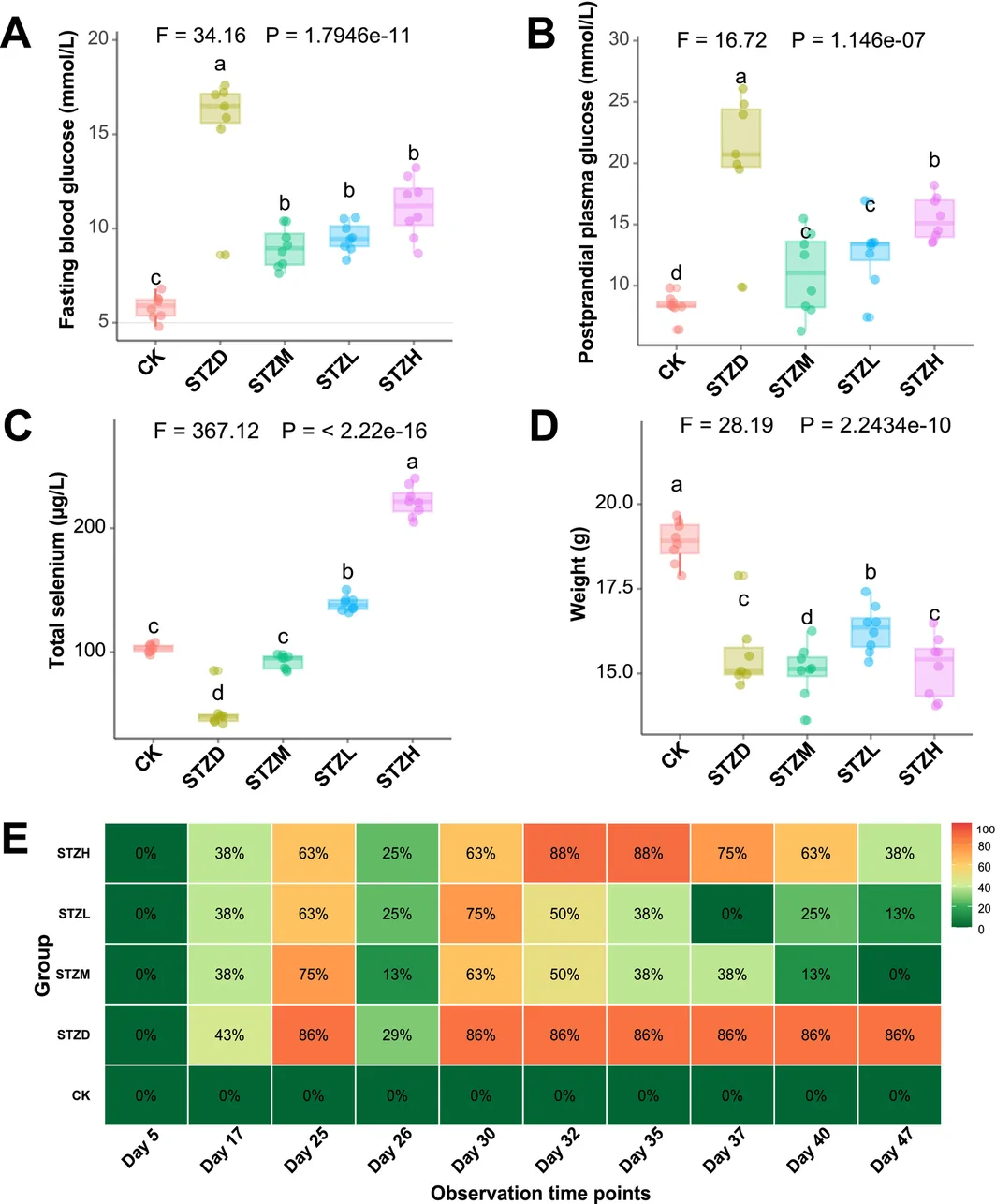
Effects of NFXBJC on fasting blood glucose, postprandial blood glucose, serum total selenium, body weight, and postprandial hyperglycemia incidence in diabetic mice. (A) Fasting blood glucose (FBG); (B) Postprandial blood glucose (PBG); (C) Serum total selenium concentrations; (D) Body weight; (E) Heatmap of postprandial hyperglycemia incidence (PBG ≥ 16.7 mmol/L) across observation time points. CK: Normal control; STZD: STZ‐induced diabetic model; STZM: STZ + metformin (200 mg/kg/day); STZL: STZ + low‐dose NFXBJC (80 mg/kg/day); STZH: STZ + high‐dose NFXBJC (400 mg/kg/day). Data are expressed as mean ± SD (*n* = 8). For (A–D), different letters indicate significant differences among groups (one‐way ANOVA followed by Tukey's HSD test, *p* < 0.05). For (E), the numbers represent the percentage of mice with PBG ≥ 16.7 mmol/L in each group at each time point.

To verify selenium bioavailability, serum total selenium levels were measured at the experimental endpoint (Figure 4C). The STZD group exhibited significantly reduced serum selenium (51.9 ± 15.2 μg/L) compared with the CK group (103.1 ± 3.6 μg/L, *p* < 0.001), indicating diabetes‐induced selenium deficiency. NFXBJC treatment dose‐dependently elevated serum selenium levels to 138.9 ± 5.7 μg/L in the STZL group and 221.8 ± 12.1 μg/L in the STZH group (both *p* < 0.001 vs. STZD), confirming efficient absorption of organic selenium from NFXBJC. The STZM group (92.4 ± 5.8 μg/L) showed no significant difference from the CK group (*p* > 0.05).

As shown in Figure 4D, the STZD group exhibited significantly lower body weight than the CK group, reflecting the catabolic state of diabetes. The STZL group maintained body weight significantly better than the STZD group and approached normal levels, whereas the STZH group showed no significant improvement.

### 3.4 Effects of NFXBJC on Blood Lipids, Renal Function, and Organ Tissues in Mice

Regarding renal function (Figure 5A,B), compared with the CK group, the STZD group had significantly elevated urea and creatinine levels (*p* < 0.001), indicating early kidney injury. The STZL group reduced both indicators to normal levels, whereas the STZH group showed a less pronounced effect.

**FIGURE 5:**
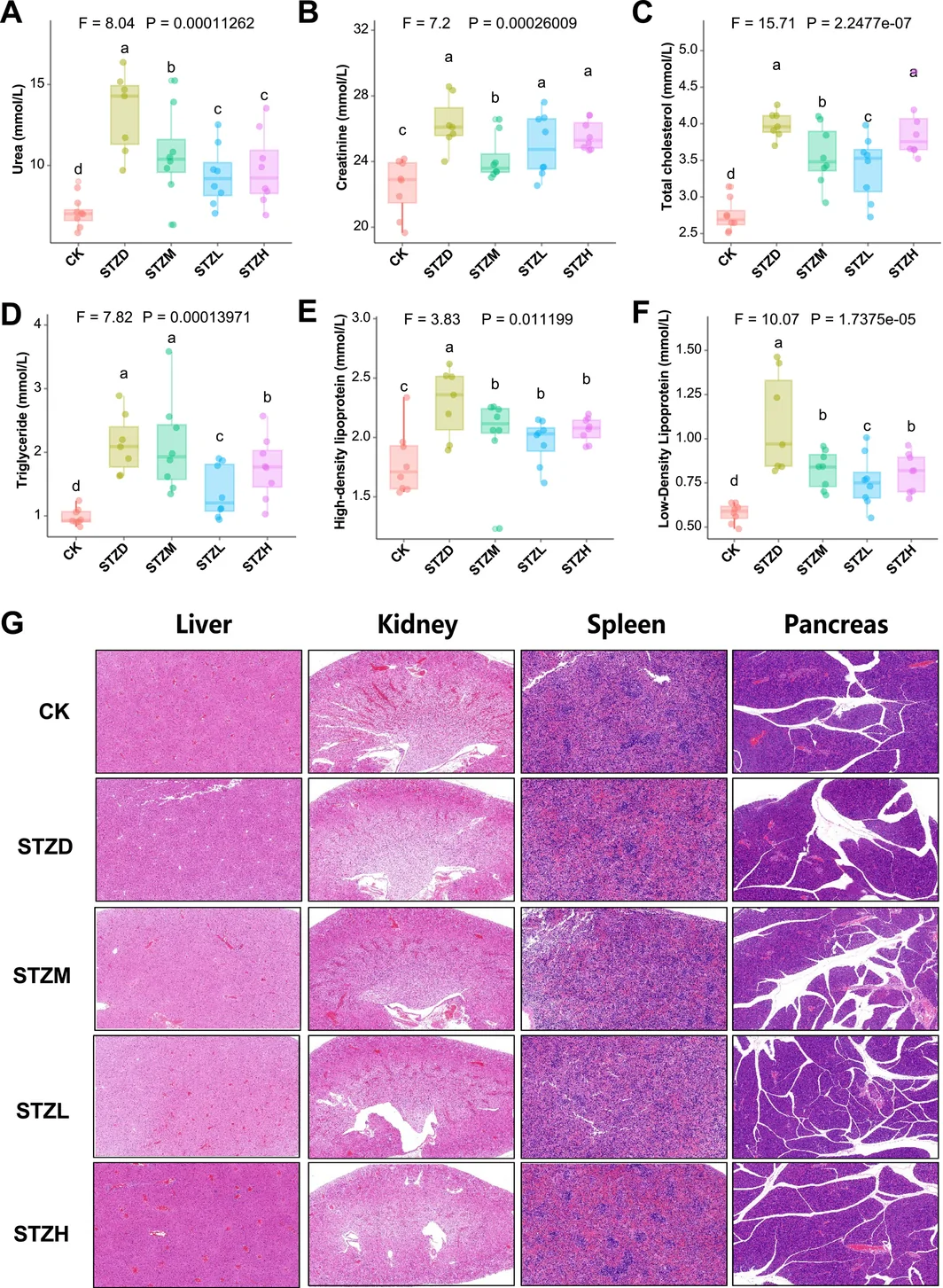
Changes in renal function, blood lipids, and organ tissues in mice after NFXBJC administration. (A) Urea; (B) Creatinine; (C) Total cholesterol; (D) Triglycerides; (E) High density lipoprotein; (F) Low density lipoprotein; (G) Hematoxylin and eosin (HE) staining of liver, kidney, spleen, and pancreas (200×). CK: Normal control group; STZD: STZ‐induced diabetes model group; STZM: STZ + metformin group (200 mg/kg/day); STZL: STZ + low‐dose NFXBJC group (80 mg/kg/day); STZH: STZ + high‐dose NFXBJC group (400 mg/kg/day). Different letters indicate significant differences between groups (*p* < 0.05), while the same letter indicates no significant difference. Data are expressed as mean ± SD (*n* = 8). Data are expressed as mean ± SD (*n* = 8). Different letters indicate significant differences among groups (one‐way ANOVA followed by Tukey's HSD test, *p* < 0.05), while the same letter indicates no significant difference.

Regarding blood lipid profiles (Figure 5C–F), the STZD group had significantly elevated TC and TG levels (*p* < 0.001), indicating typical dyslipidemia. The STZL group reduced TC and TG by 14.8% (95% CI = 0.18–1.00 mmol/L, *p* = 0.008) and 35.6% (95% CI = 0.32–1.20 mmol/L, *p* = 0.002), respectively, outperforming the STZM group. The STZH group showed less improvement than the STZL group. LDL levels showed a trend consistent with TC, whereas HDL levels showed no significant change across groups.

Histopathological examination of organ tissues (Figure 5G) showed no obvious abnormalities in the liver, kidney, spleen, or pancreas in the CK, STZM, STZL, and STZH groups. The STZD group exhibited mild histopathological changes, including hepatocellular steatosis and inflammatory cell infiltration in the liver, renal tubular injury in the kidney, white pulp hyperplasia and red pulp congestion in the spleen, and islet atrophy in the pancreas. Semi‐quantitative scoring by an observer blinded to group allocation showed that these changes were mild (mostly score 1) and that there were no significant differences among groups (*p* > 0.05) (Table S2). No obvious fatty degeneration, inflammatory infiltration, or fibrosis was observed in any group.

### 3.5 Correlation Analysis Between Blood Glucose and Serum Biochemical Indicators as Well as Body Weight in Mice

To explore associations between blood glucose levels and various metabolic indicators, Pearson linear regression was performed to analyze the relationships of FBG and PBG with serum biochemical parameters, body weight, and total selenium in mice. As shown in Figure 6A–F, both FBG and PBG were significantly positively correlated with urea (A), creatinine (B), TC (C), TG (D), high‐density lipoprotein (E), and low‐density lipoprotein (F) (all *p* < 0.01), with the strongest correlation observed for TC (FBG: *R* ^2^ = 0.405, *p* < 0.001; PBG: *R* ^2^ = 0.351, *p* < 0.001). Both FBG and PBG exhibited significant negative correlations with body weight (Figure 6G; FBG: *R* ^2^ = 0.398, *p* < 0.001; PBG: *R* ^2^ = 0.269, *p* = 0.001), indicating that higher blood glucose was associated with lower body weight, consistent with the catabolic state of diabetes.

**FIGURE 6:**
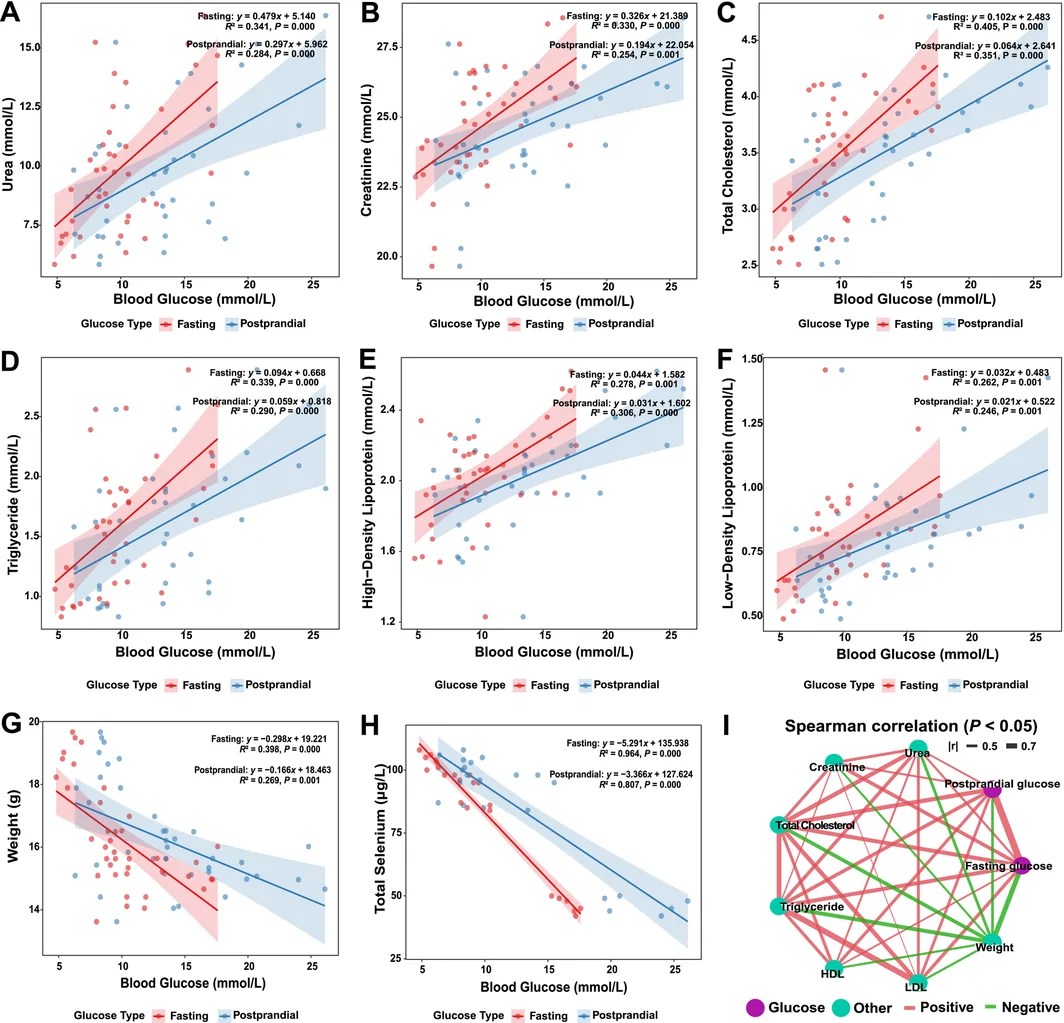
Correlation analysis between blood glucose and serum biochemical indicators, body weight, and total selenium in mice. (A) Linear regression of blood glucose with urea; (B) Linear regression of blood glucose with creatinine; (C) Linear regression of blood glucose with total cholesterol; (D) Linear regression of blood glucose with triglycerides; (E) Linear regression of blood glucose with high‐density lipoprotein; (F) Linear regression of blood glucose with low‐density lipoprotein; (G) Linear regression of postprandial blood glucose with body weight; (H) Linear regression of blood glucose with body weight; (I) Spearman correlation network plot of various indicators (only significant correlations with *p* < 0.05 are shown). Red lines indicate positive correlations, green lines indicate negative correlations, and line thickness represents the magnitude of |r|. In (A–H), *R* ^2^ and *p* values are shown in each panel.

To assess the relationship between blood glucose and selenium without interference from exogenous selenium supplementation, the analysis was restricted to the CK, STZD, and STZM groups. Under these conditions, a strong negative correlation was observed between blood glucose and serum total selenium (Figure 6H; FBG: *R* ^2^ = 0.964, *p* < 0.001; PBG: *R* ^2^ = 0.807, *p* < 0.001), indicating that hyperglycemia is closely associated with severe selenium deficiency and suggesting that diabetes itself may lead to selenium depletion.

Further Spearman correlation network analysis (Figure 6I) revealed complex associations among the indicators. Blood glucose was positively correlated with renal function and blood lipid indicators, and negatively correlated with body weight. Additionally, strong positive correlations were observed between urea and creatinine, and between TC and LDL. Collectively, these findings indicate that renal function impairment, dyslipidemia, endogenous selenium deficiency, and body weight loss are closely associated with blood glucose levels in diabetic mice.

### 3.6 Network Pharmacology Analysis

To further explore the potential mechanisms underlying the metabolic improvements observed in diabetic mice and to predict whether similar effects might occur in humans, we performed network pharmacology and molecular docking analyses based on the active ingredients of NFXBJC and human disease targets. Candidate compounds were screened through the TCMSP database, yielding 48 compounds that collectively corresponded to 552 putative targets (Table S3). These targets were intersected with disease‐associated genes for Type 2 diabetes (T2D), hyperlipidemia, and hyperuricemia. As shown in Figure 7A,E,I, the intersections yielded 508 common targets (out of 17,927 T2D targets), 386 common targets (out of 7652 hyperlipidemia targets), and 171 common targets (out of 1861 hyperuricemia targets), respectively.

**FIGURE 7:**
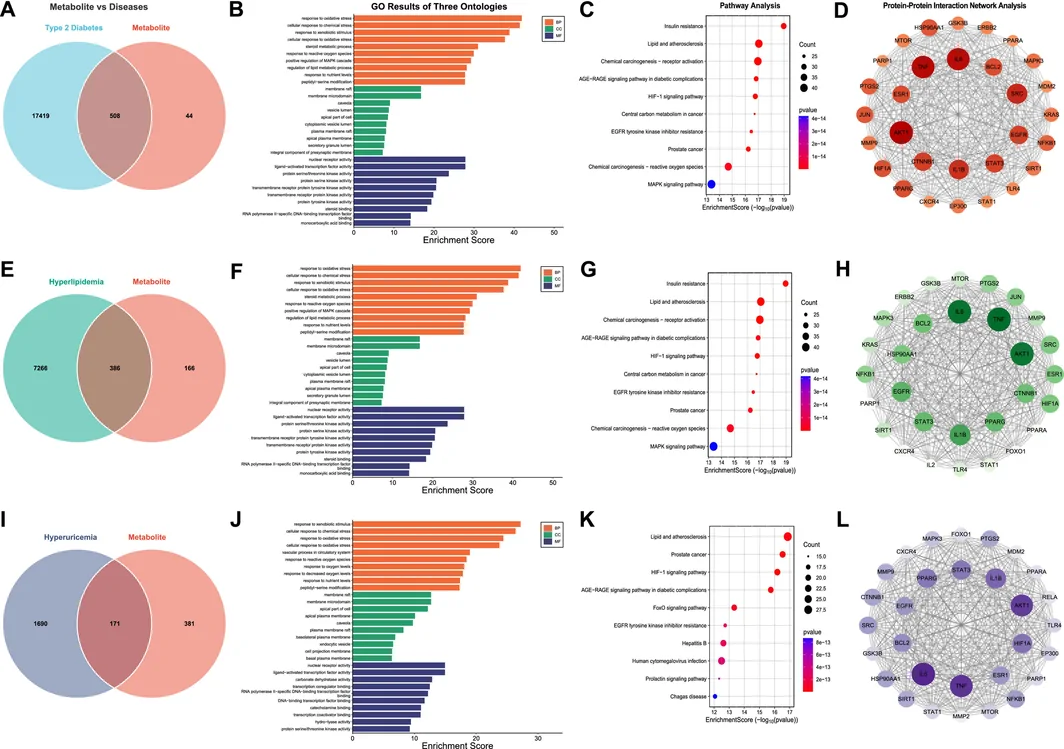
Network pharmacology analysis of NFXBJC. (A–D) T2D; (E–H) Hyperlipidemia; (I–L) Hyperuricemia. For each disease, panels show: Venn diagram of metabolite‐disease target intersection; GO enrichment analysis (BP, CC, MF); KEGG pathway enrichment analysis; and PPI network of the top 30 hub targets ranked by degree.

Gene Ontology (GO) enrichment analysis revealed that the shared targets for T2D and hyperlipidemia exhibited highly similar profiles (Figure 7B,C,F,G). The top three biological processes (BP) were response to oxidative stress, cellular response to chemical stress, and response to xenobiotic stimulus. The top three cellular components (CC) were membrane raft, membrane microdomain, and caveola, whereas the top three molecular functions (MF) were nuclear receptor activity, ligand‐activated transcription factor activity, and protein serine/threonine kinase activity. KEGG pathway analysis consistently identified insulin resistance, lipid and atherosclerosis, and chemical carcinogenesis—receptor activation as the top three pathways for both diseases. In contrast, hyperuricemia‐associated targets showed distinct features (Figure 7J,K): the third CC term was apical part of cell (instead of caveola), the third MF term was carbonate dehydratase activity (instead of protein kinase activity), and the top KEGG pathways included lipid and atherosclerosis, prostate cancer, and HIF‐1 signaling pathway, indicating a partially distinct signaling preference.

Protein–protein interaction (PPI) analysis was performed to identify core regulatory nodes. For each of the three intersection target sets, the top 30 targets ranked by degree were selected to construct interaction networks (Figure 7D,H,L). When ranked by descending degree, the top 10 hub targets were completely conserved across all three conditions, comprising STAT3, IL6, AKT1, CTNNB1, TNF, EGFR, HIF1A, IL1B, SRC, and BCL2 (Table S4). This finding suggests that, despite the differing numbers of intersecting targets (508, 386, and 171 for T2D, hyperlipidemia, and hyperuricemia, respectively), NFXBJC may exert synergistic beneficial effects on glucolipid metabolism and uric acid regulation through modulation of these shared core nodes.

### 3.7 Molecular Docking of Active Ingredients With Core Targets

To explore whether multiple active ingredients act on the same target and vice versa, we performed molecular docking between the active ingredients and the top 10 hub targets (probability ≥ 95%). Among the 10 pairs tested (Table S5), only those with binding energies below −5.0 kcal/mol were retained, yielding six pairs for further analysis. As shown in Figure 8, all six docking pairs exhibited favorable binding affinities, with binding energies ranging from −5.95 to −7.50 kcal/mol, all below the screening threshold of −5.0 kcal/mol, indicating stable potential interactions between these compounds and the target proteins. Among them, cryptotanshinone bound to STAT3 with the lowest binding energy of −7.50 kcal/mol (Figure 8A), suggesting the strongest binding affinity for this target. Isorhamnetin displayed multi‐target binding capability, with binding energies of −6.04 kcal/mol for AKT1 (Figure 8B) and −6.24 kcal/mol for SRC (Figure 8C). For AKT1, multiple active ingredients also demonstrated effective binding, with kaempferol showing a binding energy of −5.95 kcal/mol (Figure 8D), morin −6.12 kcal/mol (Figure 8E), and quercetagetin −6.57 kcal/mol (Figure 8F). All complexes were stabilized by hydrogen bonds, hydrophobic interactions, and van der Waals forces, among which hydrogen bonds played a critical role in anchoring the ligands within the active pockets. For instance, isorhamnetin formed multiple hydrogen bonds with AKT1 residues such as THR‐211 and TYR‐263, whereas quercetagetin established polar contacts with THR‐87, ARG‐15, and ASP‐274 of AKT1, further stabilizing the ligand–protein complexes.

**FIGURE 8:**
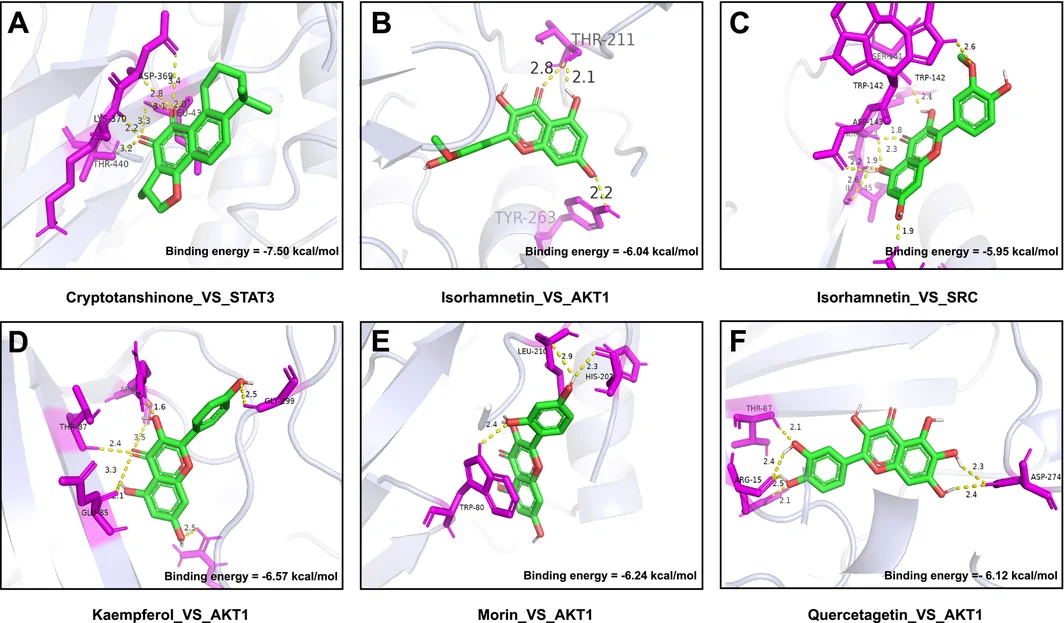
Molecular docking of core active ingredients from NFXBJC with hub targets. (A) Cryptotanshinone with STAT3; (B) Isorhamnetin with AKT1; (C) Isorhamnetin with SRC; (D) Kaempferol with AKT1; (E) Morin with AKT1; (F) Quercetagetin with AKT1. Binding energies are indicated below each panel. Hydrogen bonds are shown as yellow dashed lines, and interacting residues are labeled accordingly.

### 3.8 Exploratory Human Observation of Metabolic Improvements

To preliminarily validate whether the multi‐target effects predicted by network pharmacology could translate into clinical benefits, we conducted an exploratory small‐scale human observational study in individuals with hyperglycemia, hyperlipidemia, and/or hyperuricemia. As shown in Figure 9, in the placebo control group W, uric acid levels remained elevated (390–420 μmol/L) with no significant change over 120 days (*p* > 0.05), whereas FBG and TC remained within normal ranges without notable fluctuation. In contrast, NFXBJC treatment led to significant improvements in groups X, Y, and Z. Group X (hyperuricemic) showed progressive uric acid reduction from Day 15 onward, accompanied by moderate decreases in FBG and TC. Group Y (combined hyperglycemia, hyperlipidemia, and hyperuricemia) exhibited the most robust response, with all three indicators declining continuously from baseline to Day 120 (*p* < 0.05). Group Z (hyperuricemic) also demonstrated significant uric acid reduction, along with a delayed but effective FBG‐lowering effect and a consistent TC decrease. These results indicate that NFXBJC effectively lowers uric acid, blood glucose, and total cholesterol in a time‐dependent manner, with the most pronounced effect on uric acid reduction, whereas untreated hyperuricemic controls showed no spontaneous improvement. Given the very limited sample size (*n* = 4 subjects) and the exploratory, case‐based design, no formal between‐group statistical comparison was performed, and these findings should be considered preliminary and hypothesis‐generating only.

**FIGURE 9:**
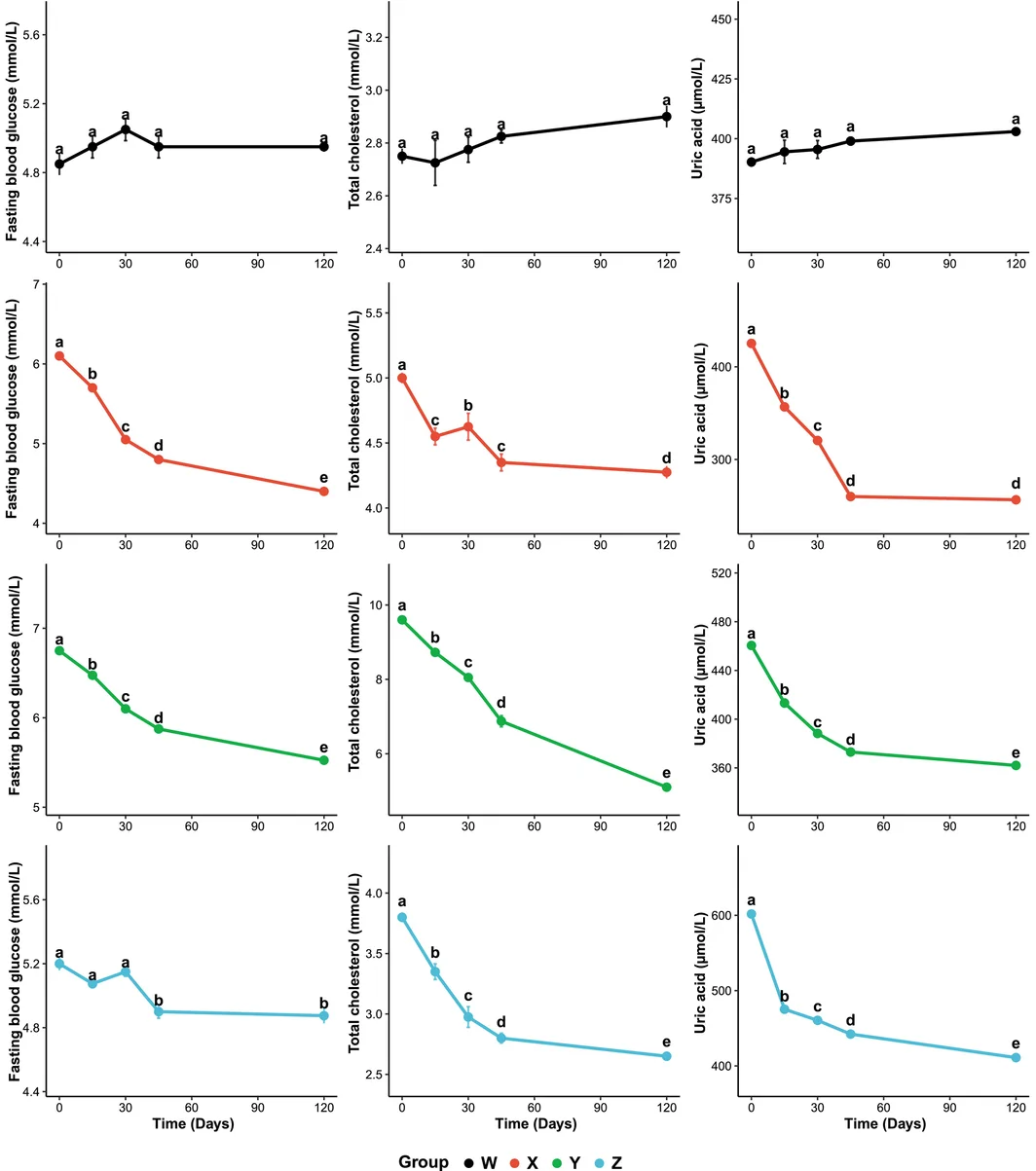
Changes in blood glucose, total cholesterol, and uric acid levels in subjects before and after NFXBJC administration. Group W was the placebo control (receiving a maltodextrin‐based placebo, dissolved in warm water); groups X, Y, and Z received NFXBJC for 120 days. Different letters indicate significant differences between time points within the same group (*p* < 0.05). Data are expressed as mean ± SD (*n* = 4).

## 4 Discussion

### 4.1 Active Component and Metabolomic Profiling for Formulation Selection

The two candidate formulations were designed based on two distinct dietary patterns identified among centenarians in Enshi City. OFXBJC reflects a diet richer in edible mushrooms, whereas NFXBJC reflects a diet richer in medicinal plants such as *Cyclocarya paliurus* and *Polygonatum sibiricum*. This fundamental compositional difference accounts for the marked variations in their active components and metabolic profiles.

Total flavonoids, total saponins, and soluble protein contents were significantly higher in NFXBJC than in OFXBJC. This is attributable to the compositional features of NFXBJC, which incorporated increased proportions of *Lentinula edodes*, *Flammulina filiformis*, and *Boletus edulis*, along with the addition of *Cyclocarya paliurus* and *Polygonatum sibiricum*. *Cyclocarya paliurus* is rich in triterpenoids and flavonoids, and *Polygonatum sibiricum* contains steroidal saponins, directly contributing to the increases in total flavonoids and total saponins (Liu et al. 2018; Tang et al. 2019). The higher soluble protein content may be attributable to the combined contribution of the added *Polygonatum sibiricum* and the increased proportions of protein‐rich mushrooms such as *Lentinula edodes*, *Flammulina filiformis*, and *Boletus edulis* (Ionescu et al. 2025). Metabolomics analysis further revealed a clear separation in the metabolic profiles between OFXBJC and NFXBJC, with far more upregulated than downregulated metabolites in NFXBJC. Hypoglycemic active metabolites such as corosolic acid and various flavonoids were significantly increased. Additionally, the elemental selenium content of NFXBJC (37.4 mg/kg) was higher than that of OFXBJC (30.0 mg/kg). Based on these comprehensive comparisons, including higher bioactive components, a more favorable metabolomic profile, and higher selenium content, NFXBJC was selected as the optimized formulation for subsequent in vivo efficacy studies (Sundararajan et al. 2014; Gazwi et al. 2023).

### 4.2 Efficacy Evaluation of NFXBJC in Mice

The animal experiment showed that the STZL group had significant effects in reducing FBG and PBG and performed best in maintaining body weight. Interestingly, the beneficial effects were not proportional to the dose, as the low‐dose group consistently outperformed the high‐dose group in improving blood glucose, blood lipids, and renal function. This nonlinear dose–response pattern may be explained by several factors. First, the abundant polysaccharides in NFXBJC may contribute to this effect. Moderate polysaccharide intake has been reported to exert hypoglycemic effects, whereas excessive intake may alter intestinal osmotic pressure or gut microbiota and thereby affect nutrient absorption and glycemic stability (Chen et al. 2022). In addition, high doses of certain active ingredients could possibly lead to antagonistic effects or metabolic saturation, although this remains speculative (Jamal et al. 2024). Second, selenium is well known to exhibit a U‐shaped dose–response, in which moderate supplementation confers benefits while excessive intake may diminish efficacy (Chang et al. 2025). In the present study, although serum selenium increased dose‐dependently, the STZH group did not show better glycemic improvement than the STZL group, which is consistent with this U‐shaped pattern. Taken together, these factors suggest that the STZL group may represent a better balance between selenium replenishment and glycemic control, but further dose–response studies are needed to confirm this.

The postprandial hyperglycemia incidence further supported the advantage of the STZL group, which achieved an 84.8% reduction compared with the STZD group. Regarding blood lipids and renal function, the STZL group also demonstrated significant improvements. It reduced TC and TG by 21.8% and 34.6%, respectively, decreased LDL, and restored urea and creatinine to normal levels. Diabetic kidney disease (DKD) is characterized by elevated urea and creatinine (Butt et al. 2024), and the reversal of these abnormalities by STZL indicates effective renoprotection. Correlation and network analyses further revealed that blood glucose was positively correlated with renal and lipid indicators and negatively correlated with body weight, suggesting close interactions among glucose metabolism, renal function, and dyslipidemia (Rong et al. 2024; Qin et al. 2026).

Notably, the STZD group exhibited severe selenium deficiency compared with the CK group, indicating that diabetes itself may lead to selenium depletion (Navarro‐Alarcon et al. 1999). NFXBJC treatment dose‐dependently replenished serum selenium levels, confirming efficient absorption of organic selenium from the product. Correlation analysis restricted to groups without exogenous selenium intervention revealed a strong negative association between blood glucose and serum selenium, further supporting the link between hyperglycemia and selenium deficiency (Wang et al. 2020).

Finally, histopathological examination showed no obvious abnormalities in the liver, kidney, spleen, or pancreas of any group, indicating that NFXBJC has good safety at the tested doses.

### 4.3 Network Pharmacology and Molecular Docking

Computational approaches combined with in vitro assays have been increasingly used to explore the multi‐target potential of plant‐derived bioactive compounds (Fatima et al. 2025). To gain mechanistic insights into the simultaneous improvements in hyperglycemia, hyperlipidemia, and hyperuricemia observed in our exploratory human observational study, we performed network pharmacology analysis based on human disease targets. From the 48 candidate compounds identified in NFXBJC, a total of 552 putative targets were predicted. Intersection with disease‐associated genes from the GeneCards database yielded 508 common targets for Type 2 diabetes (T2D), 386 for hyperlipidemia, and 171 for hyperuricemia. Protein–protein interaction (PPI) analysis revealed that the top 10 hub targets ranked by degree, namely STAT3, IL6, AKT1, CTNNB1, TNF, EGFR, HIF1A, IL1B, SRC, and BCL2, were completely conserved across all three conditions. This striking conservation suggests that NFXBJC may exert its pleiotropic beneficial effects through modulation of a shared “inflammation–metabolism” core network rather than through entirely disease‐specific pathways.

GO and KEGG enrichment analyses further supported this interpretation. The top biological processes included response to oxidative stress and cellular response to chemical stress, while KEGG pathways were prominently enriched in insulin resistance, lipid and atherosclerosis, and HIF‐1 signaling. These pathways are closely interconnected with the clinical parameters measured in our human study. For instance, IL‐6 and TNF are well‐established pro‐inflammatory cytokines that promote insulin resistance and impair renal uric acid excretion via modulation of organic anion transporters (Sharma and Kanneganti 2021; Han 2025). AKT1 is a central node of the PI3K/AKT insulin signaling cascade that regulates both glucose uptake and lipogenesis (Verma et al. 2023). STAT3 functions dually as an inflammatory transducer and a transcriptional regulator of lipid metabolism (Wang, Hu, et al. 2024; Wang, Li, et al. 2024). The convergence of these targets onto a single conserved network offers a mechanistic explanation for why a multi‐component formulation such as NFXBJC could simultaneously improve hyperglycemia, hyperlipidemia, and hyperuricemia in the same individuals, a pattern clearly observed in subject Y.

To further predict whether the core active ingredients of NFXBJC can physically interact with these hub targets, we performed molecular docking using AutoDock Vina. All six representative compound–target pairs tested exhibited binding energies below −5.0 kcal/mol, ranging from −5.95 to −7.50 kcal/mol, indicating favorable and stable protein–ligand interactions. Among them, cryptotanshinone showed the strongest binding affinity to STAT3 at −7.50 kcal/mol, suggesting potent potential for modulating this inflammatory signaling node. Notably, the docking results revealed a multi‐dimensional interaction pattern. On one hand, isorhamnetin exhibited multi‐target binding capability, with binding energies of −6.04 kcal/mol for AKT1 and −6.24 kcal/mol for SRC, exemplifying the “one‐compound, multiple‐targets” property commonly observed in natural flavonoids (Hao et al. 2025; Luo et al. 2026). On the other hand, multiple flavonoids, including kaempferol (−5.95 kcal/mol), morin (−6.12 kcal/mol), and quercetagetin (−6.57 kcal/mol), in addition to isorhamnetin, effectively bound to the same target AKT1, demonstrating a “multiple‐compounds, one‐target” synergistic mode (Wang, Liu, et al. 2024; Wang, Li, et al. 2024). This polypharmacological profile provides preliminary structural evidence suggesting the holistic regulatory advantage of NFXBJC's multi‐component formula in addressing the interconnected pathologies of metabolic disorders.

Taken together, the network pharmacology and molecular docking analyses suggest that the beneficial effects of NFXBJC on hyperglycemia, hyperlipidemia, and hyperuricemia may be attributable, at least in part, to the synergistic modulation of a conserved set of hub targets that bridge inflammation and metabolism, providing a mechanistic framework for the pleiotropic actions of this multi‐component formulation.

### 4.4 Exploratory Human Observation Provides Translational Evidence

To preliminarily validate whether the multi‐target effects predicted by network pharmacology could translate into clinical benefits, we conducted an exploratory small‐scale human observational study in individuals with hyperglycemia, hyperlipidemia, and/or hyperuricemia. Consistent with the animal experiment and in silico predictions (Xiao et al. 2022), NFXBJC administration for 120 days progressively reduced uric acid, blood glucose, and total cholesterol. Notably, subject Y, who presented with combined hyperglycemia, hyperlipidemia, and hyperuricemia, showed simultaneous improvements in all three parameters, mirroring the overlapping target network shared across these conditions. The most pronounced effect was observed for uric acid reduction (32.2%–39.8%), which aligns with the enrichment of HIF‐1 signaling and inflammatory pathways identified in the hyperuricemia intersection targets (Kimura et al. 2016; Huang et al. 2023). The lack of improvement in the placebo control (subject W) ruled out spontaneous remission or dietary confounding as major contributors.

However, several limitations of this exploratory human observational study should be acknowledged. First, the small sample size (*n* = 4) precludes statistical generalization, and the results should be interpreted as preliminary evidence rather than definitive proof of efficacy. Second, the human study did not measure serum selenium levels, which limits our ability to confirm whether the selenium‐replenishing effect observed in mice also occurs in humans. Third, as a computational prediction, network pharmacology analysis does not replace experimental validation; future studies should employ targeted approaches such as ELISA, Western blot, or cellular assays to verify the involvement of the identified hub targets, including IL‐6, TNF, and AKT1, in the mechanism of action of NFXBJC. Fourth, the candidate compounds were identified through untargeted metabolomics combined with TCMSP screening; a comprehensive list of these 48 compounds, with their corresponding sources and OB/DL values, is provided in Table S3. Despite these limitations, the consistent time‐dependent improvements across multiple metabolic indicators in the exploratory human observational study, together with the conserved hub targets identified by network pharmacology, provide preliminary human observational and mechanistic evidence supporting the translational potential of NFXBJC as a functional food for metabolic health. Future randomized controlled trials with larger cohorts and mechanistic biomarkers are warranted to validate these findings.

## 5 Conclusion

Inspired by the mushrooms and plants consumed by centenarians in Enshi City, two products, OFXBJC and NFXBJC, were designed and compared. NFXBJC, with higher levels of total flavonoids, total saponins, soluble protein, and hypoglycemic‐related metabolites, was selected for subsequent efficacy studies. In diabetic mice, NFXBJC dose‐dependently replenished selenium, while low‐dose NFXBJC (80 mg/kg/day) significantly reduced blood glucose, blood lipids, urea, and creatinine, improved early kidney injury, and showed good safety. Correlation analysis revealed a strong negative association between blood glucose and selenium, suggesting that diabetes itself may lead to selenium depletion. Network pharmacology and molecular docking analyses further elucidated the multi‐target mechanisms of NFXBJC, revealing that its active ingredients modulate a conserved set of 10 hub targets (STAT3, IL6, AKT1, CTNNB1, TNF, EGFR, HIF1A, IL1B, SRC, and BCL2) bridging inflammation and metabolism across diabetes, hyperlipidemia, and hyperuricemia, with stable protein–ligand interactions predicted by docking. An exploratory small‐scale human observational study preliminarily suggested that NFXBJC may improve hyperuricemia, hyperglycemia, and hyperlipidemia, with the most pronounced effect on uric acid reduction. However, given the very limited sample size, these findings are hypothesis‐generating only and should not be interpreted as clinical conclusions. In summary, NFXBJC may have potential as a functional food for improving blood glucose, blood lipids, and uric acid, as well as replenishing selenium, through multi‐target synergistic mechanisms, but larger randomized controlled trials are needed to confirm these effects.

## Author Contributions

**Wuxian Zhou:** conceptualization, methodology, software, data curation, investigation, validation, formal analysis, visualization, writing – original draft, writing – review and editing. **Fanfan Wang:** methodology, software, data curation, investigation. **Yuanyuan Duan:** investigation, validation, formal analysis, data curation, software. **Xiaogang Jiang:** methodology, software, data curation, investigation, validation. **Hongchang Xiao:** funding acquisition, project administration, resources. **Aimin Yu:** funding acquisition, project administration, resources. **Hui Kuang:** validation, software, methodology, investigation. **Bo Zhu:** methodology, software, validation, investigation. **Qingfang Wang:** conceptualization, writing – review and editing, project administration, supervision, funding acquisition. **Jingmao You:** funding acquisition, conceptualization, writing – review and editing, project administration, supervision.

## Funding

This work was supported by the China Agriculture Research System (CARS‐20‐202640), Hubei Key Research and Development Program (2023BBB079), Hubei Natural Science Foundation Project (2025AFD159), and Enshi Technology Research Project (Qihang Special Project, 33).

## Ethics Statement

The animal experiment was approved by the Laboratory Animal Ethics Committee of the Institute of Chinese Herbal Medicines, Hubei Academy of Agricultural Sciences (Approval No. ICHM‐A‐2025002). The human study was approved by the Ethics Committee of the Institute of Chinese Herbal Medicines, Hubei Academy of Agricultural Sciences (Approval No. ICHM‐H‐2025001), and all participants provided written informed consent.

## Conflicts of Interest

The authors declare no conflicts of interest.

## Supporting information


**Table S1:** Extraction yields of individual raw materials and the complete formulations of OFXBJC and NFXBJC.

**Table S2:** Semi‐quantitative histopathological scores of liver, kidney, spleen, and pancreas in mice.

**Table S3:** Chemical information of the 48 candidate compounds identified from NFXBJC.

**Table S4:** Top 30 hub targets ranked by degree centrality for Type 2 diabetes, hyperlipidemia, and hyperuricemia.

**Table S5:** Molecular docking probabilities of core active compounds against the top 10 shared hub targets.

## Data Availability

The data that support the findings of this study are available on request from the corresponding author. The data are not publicly available due to privacy or ethical restrictions.
